# Supplementary material for: The potential of tumour microenvironment markers to stratify the risk of recurrence in prostate cancer patients
Source: PLoS One. 2020 Dec 28;15(12):e0244663. doi: 10.1371/journal.pone.0244663 (PMC7769484; doi:10.1371/journal.pone.0244663)
Supplement: S1 Table — (DOCX) [file pone.0244663.s001.docx]

**S1 Table: Clinical and pathological characteristics of the LR and HR groups**

|  | | **Total** | | | | **LR** | | | **HR** | | **P-value** | | |  |  |
| --- | --- | --- | --- | --- | --- | --- | --- | --- | --- | --- | --- | --- | --- | --- | --- |
|  | | **Med** | | **IQR** | | **Med** | **IQR** | **Med** | | **IQR** | |  | | |  |
| **Age (years)** | | 65 | | 61-72 | | 67.5 | 59-71 | 65 | | 61-72 | | 0.88 | | |  |
|  | | **No.** | |  | | **No.** |  | **No.** | |  | |  | | |  |
| **Tumor stage** | |  | |  | |  |  |  | |  | | NA | | |  |
| T2 | | 8 | |  | | 4 |  | 4 | |  | |  | | |  |
| T3a | | 18 | |  | | 7 |  | 11 | |  | |  | | |  |
| T3b | | 8 | |  | | 3 |  | 5 | |  | |  | | |  |
| T4 | | 1 | |  | | 0 |  | 1 | |  | |  | | |  |
| **Nodal stage** | | |  | |  | |  |  | |  | | | NA | | |
| Negative (N0) | 34 | |  | | 14 | |  | 20 | |  | | |  | | |
| Positive (N+) | 1 | |  | | 0 | |  | 1 | |  | | |  | | |
| **ISUP Grade Group** | | |  | |  | |  |  | |  | | | 0.74 | | |
| **GG2** | 14 | |  | | 5 | |  | 9 | |  | | |  | | |
| **GG3** | 21 | |  | | 9 | |  | 12 | |  | | |  | | |
| **Surgical margins** | | |  | |  | |  |  | |  | | | 0.70 | | |
| Positive | 10 | |  | | 3 | |  | 7 | |  | | |  | | |
| Negative | 25 | |  | | 11 | |  | 14 | |  | | |  | | |
| **Total** | 35 | |  | | 14 | |  | 21 | |  | | |  | | |

The p-values were computed using the Mann-Whitney test for age and the exact Fisher test for the binary features.
