# Supplementary material for: The potential of tumour microenvironment markers to stratify the risk of recurrence in prostate cancer patients
Source: PLoS One. 2020 Dec 28;15(12):e0244663. doi: 10.1371/journal.pone.0244663 (PMC7769484; doi:10.1371/journal.pone.0244663)
Supplement: Attachment — Submitted filename: Answer to the referees.docx [file pone.0244663.s002.docx]

We thank the reviewers and editor for the interesting and adequate comments and we have carefully answered and addressed all of the reviewers’ and editors’ comments.

\# Editor:

**1. Please ensure that your manuscript meets PLOS ONE's style requirements, including those for file naming. The PLOS ONE style templates can be found at**

Answer: Done.

**2. In the ethics statement in the manuscript and in the online submission form, please provide additional information about the patient samples used in your retrospective study, including: a) whether all data were fully anonymized before you accessed them; b) the date range (month and year) during which patients' medical records/samples were accessed and/or whether the IRB or ethics committee waived the requirement for informed consent; c) the date range (month and year) during which** **patients whose medical records/samples were selected for this study sought treatment; and d)** **the source of the medical records/samples analyzed in this work (e.g. hospital, institution or medical center name). If patients provided informed written consent to have data from their medical records used in research, please include this information.**

Answer: Done.

**3. Please note that PLOS does not permit references to “data not shown” or "not described" Authors should provide the relevant data within the manuscript, the Supporting Information files, or in a public repository. If the data are not a core part of the research study being presented, we ask that authors remove any references to these data.**

Answer: We have included a supplementary table S1.

**4. At this time, we ask that you please provide scale bars on the microscopy images presented in Figures 1, 2 and 4, and refer to the scale bar in the corresponding Figure legend**

Answer: Scale bars (50µm) for figures 1, 2 and 4 are added and referred to in the corresponding figure legends.

\# Reviewer 1:

**1. English language:**

Answer: The manuscript had been revised for English grammar and adapted accordingly.

**2. Materials and Methods: line 135. Are the methods similar or the same? Please improve this sentence and add information:**

Answer: This has been addressed in the revised manuscript.

**3. Results: In table 1 is not visible the Gleason's score, but the Gleason's grade, while in the paragraph "Marker expression profiles in PCa versus PNT and in stroma versus epithelium" the authors cite the Gleason's score. Please add information to the table or make everything more homogeneous and clear.**

Answer: This should be: ‘ISUP Grade Group’ and has been changed accordingly. ‘ISUP Grade Group ‘ and Gleason Score are the two accepted terminologies to categorize prostate cancer grades.

**4. Is there also an important marker for TME, in particolar for Carcinoma associated fibroblasts. It is FAP-1. Have the authors evidences about its expression?**

Answer: This is an interesting remark form the reviewer. We did not check this one (also since this study has been designed with a similar marker panel as previously used (Gevaert el al. Scientific Reports, 2018). We will consider including FAP-1 in future research projects.

**5. The authors should add a table resuming the first paragraph of the results and that could support the figure 1, also specifying if the proteins analyzed are present in the epithelial or the stromal compartments.**

Answer: A table has been added accordingly in the revised manuscript (Table 3).

**6. The authors talk about the importance of TME in prostate cancer. They should take also in consideration the importance of steroid hormones receptors expression in this compartment, discussing about their absence/presence. They could consider for example: 1)** [**https://doi.org/10.3389/fendo.2014.00225**](https://doi.org/10.3389/fendo.2014.00225)**;) Endocrine-Related Cancer
(2018) 25, R179–R196 and therein refs.**

Answer: The possible role of stromal steroid hormone receptors in PCa progression is addressed in the discussion section and we also have referenced the proposed paper (and others).

**7. In general, the authors should improve the manuscript, the tables, the English grammar for enlarging the audience and rendering the manuscript more fluent.**

Answer: The manuscript has been revised and improved, tables have been improved + new tables are included , and the paper has been revised for English grammar.

Reviewer #2:

**1. Introduction:**

**The biomarkers discussed in the intro deal with predictive markers for treating metastatic disease and have nothing to do with the work presented. Biomarker panels (oncotype, decipher, prolaris, etc) for prognosticating recurrence are discussed in the same referenced paper in the intro and do deal with the same types of cases presented here and this should really be the focus of the intro. In fact, the impetus for doing a study like this would be to develop a significantly cheaper panel for doing the same job as these panels do, but at a significantly cheaper price. Also, this should be extended to the discussion since all of these panels also include stromal (TME) markers within the panels, so how this IHC panel does or does not include these /compare to what is in commercial panels would be very informative.**

Answer:

The introduction and discussion sections have been revised and amended accordingly. The commercially available prognostic biomarker panels are discussed related to our panel and related to the pro’s and con’s of IHC.

**2. Methods:**

**- TMA core size should be indicated (.6mm, 1mm or other?):**

Answer: 6mm, changed accordingly.

**- It is unclear how many cores of tumor per case and normal per case were included in the TMAs and it is mentioned that there were 6 cores taken and 3 TMAs made, but unclear if it is 3 copies of the same TMA design or that it took 3 sperate TMAs to get all of the cores included? 6 cores with three tumor / three normal? Other combo? Unclear, please clarify. While these details may seem mundane, there are some investigators that believe that at least 3 tumor cores are needed per case to account for tumor heterogeneity (for example see Rubin, Pienta, et all from around 2000 or 2002 for details), although a sentence of why such triplicates are not needed for this study is also acceptable.**

Answer: Three TMAs were required to include all cores from the 48 patients. Per patient, 6 tumor cores and 6 normal tissue cores were included. This has been clarified in the revised manuscript.

**- Also, as the makeup of the stroma might be different depending on where in the normal it is taken from (peripheral, central, transistional, periurethral, how close or far from the tumors), was there any effort to be consistent about where the normal was sampled from??? How close or far from the tumor was the normal taken? This should be included somewhere since there is discussion of the tumor field effect in discussion section.**

Answer: Since almost all tumours (ISUP GG2-3) were peripheral zone tumours, control tissue was taken from the contralateral non-tumour peripheral zone. This has been added to the methods section.

**3. Table 1, you really should have p values for each to be prognostic of clinical recurrence, because that would indicate whether clinical variables alone are prognostic and then whether the cohort needs to be reconsidered/redesigned.**

Answer: The p-values for evaluating the prognostic impact were added in Table 1. Because of the matched cohorts, p-values > 0.05 were expected.

**4. In relation to this would be something about the details of the CLRs, how many were distant (bone or soft tissue mets) vs. how many were local failures (prostate bed or local LN mets), the latter of which might be more a function of surgical technique rather than actual tumor biology. Including such information could further help the reader put these results into perspective.**

Answer: These details on CLRs are now included in the methods section. Of note, patient groups were matched for section margins and lymph node status, parameters which are likely to contribute to CLR (see also previous comment).
